# Reproducibility of tailored and universal nonselective excitation pulses at 7 T for human cardiac MRI: A 3‐year and an interday study

**DOI:** 10.1002/mrm.30495

**Published:** 2025-03-13

**Authors:** Manuel Fernando Sánchez Alarcón, Sebastian Dietrich‐Conzelmann, Jean Pierre Bassenge, Jeanette Schulz‐Menger, Sebastian Schmitter, Christoph Stefan Aigner

**Affiliations:** ^1^ Physikalisch‐Technische Bundesanstalt (PTB) Braunschweig and Berlin Germany; ^2^ Experimental and Clinical Research Center (ECRC), A Cooperation Between the Charité Medical Faculty and the Max‐Delbrück Center for Molecular Medicine and HELIOS Hospital Berlin Buch Berlin Germany; ^3^ German Center for Cardiovascular Research (DZHK), Partner Site Berlin Berlin Germany; ^4^ Helios Clinics Berlin‐Buch Department of Cardiology and Nephrology Berlin Germany; ^5^ Medical Physics in Radiology German Cancer Research Center (DKFZ) Heidelberg Germany; ^6^ Center for Magnetic Resonance Research University of Minnesota Minneapolis Minnesota USA; ^7^ Max Planck Research Group MR Physics Max Planck Institute for Human Development Berlin Germany

**Keywords:** 7 Tesla, cardiac MRI, parallel transmission, universal pulse

## Abstract

**Purpose:**

Ultrahigh‐field (UHF; ≥7 T) MRI is challenging due to spatially heterogeneous B_1_^+^ profiles. This longitudinal study evaluates the reproducibility of three parallel‐transmission excitation strategies to enable UHF cardiac MRI: vendor‐supplied radiofrequency (RF) shim, subject‐tailored kT‐points pulses (TPs), and universal kT‐points pulses (UPs).

**Methods:**

Six healthy subjects underwent 7 T MRI scans performed by different MR operators using a 32‐element parallel‐transmission body array at four time points over 3 years. A single UP was computed and applied to all subjects. TPs were computed individually for each scan and organized into four configurations. Each configuration was applied to all scans from each subject to analyze intrasubject variability. Reproducibility was assessed by comparing the coefficient of variation (CV) of simulated flip angles (FAs) within the heart volume across scan sessions.

**Results:**

TPs designed for a specific scan session yielded lower CVs (2‐fold reduction) than UP. Applying TPs to other scan sessions of the same subject, however, resulted in approximately 40% higher CVs and lower FA uniformity compared with the UP. On average, the UP consistently achieved the most reproducible results across inter‐year, inter‐day, and same‐operator studies, with CVs of approximately 12%.

**Conclusion:**

Although TPs showed advantages when tailored for a specific target volume, they struggled with long‐term consistency and required lengthy calibration. The precomputed UP kT‐points pulses proved to be the most consistent across all scans acquired in the 3 years by different operators, minimizing CV‐data dispersion and maintaining FA uniformity.

## 1 INTRODUCTION

Ultrahigh field (UHF) ≥ 7 T MRI offers multiple advantages compared with lower field strengths, including enhanced signal‐to‐noise ratio, spectral resolution, and, for specific applications such as T_2_*‐weighted imaging, a higher contrast. Consequently, UHF MRI substantially advances many applications in clinical and research contexts.^1^ Notably, it plays a vital role in neuroimaging, facilitating detailed exploration of brain structures and functional connectivity,^2^, ^3^ and it has proven valuable in cardiac imaging.^4^, ^5^, ^6^, ^7^, ^8^ In particular, UHF cardiac imaging has the potential to fill a critical gap in spatiotemporal resolution crucial for understanding myocardial and pathological processes.^8^

However, UHF MRI faces limitations due to inhomogeneities in the transmit (Tx) electromagnetic radiofrequency (RF) field ($B_{1}^{+}$), resulting in spatially variable flip angles (FAs), with a possible presence of FA dropouts, which can affect both quantitative evaluations and image‐based diagnoses.^1^, ^9^ Several strategies have emerged to tackle this challenge, including the use of adiabatic RF pulses,^10^, ^11^ dielectric pads,^12^, ^13^ dedicated RF coil designs,^14^, ^15^ and parallel transmission (pTx),^16^, ^17^, ^18^, ^19^, ^20^, ^21^ the latter being regarded as the most flexible approach.^16^ In addition, UHF cardiac MRI encounters notably significant technical hurdles originating from various sources of motion, such as respiration, cardiac activity, and blood flow and requires complex and lengthy $B_{1}^{+}$ adjustment and pTx optimization routines.^22^, ^23^, ^24^, ^25^

Subject‐tailored static pTx (or RF shimming) and dynamic pTx using kT‐points emerge as a highly viable option for reducing FA heterogeneities, offering a practical balance between FA heterogeneity mitigation and RF power for cardiac applications at 7 T.^25^, ^26^ Nevertheless, while calibration times as brief as 30 s are achievable for human brain applications including channel‐wise three‐dimensional (3D) multislice absolute $B_{1}^{+}$ mapping,^27^ the adjustment time of tailored RF pulses (TP) in the human body is considerably longer and can exceed 10 min, depending on the resolution and the chosen technique, due to the lack of online adjustment methods at our MR console and the need for motion‐robust $B_{1}^{+}$ mapping.^22^, ^25^, ^28^ However, the issue of long adjustment times has been successfully addressed using calibration‐free approaches.^22^, ^28^ This concept was initially introduced for the human brain at 7 T by Gras et al.^29^ using the so‐called universal RF pulses (UP) and was extended for the heart^28^ and spinal cord.^30^, ^31^ UPs facilitate faster and simpler 7T cardiac imaging with a streamlined workflow, potentially enabling clinical and clinical research applications such as UHF four‐dimensional flow imaging.

Previous studies have delved into the advantages of TP and UP pTx for cardiac applications, primarily concentrating on immediate enhancements in FA homogeneity and RF power efficiency.^22^, ^25^, ^26^, ^28^, ^32^ However, a critical gap persists: the lack of rigorous examination of the reproducibility of the RF excitation performance over time. The question of how the coil position and MRI operators affect TP and UP pTx remains unanswered. Furthermore, it is unclear whether TP and UP pTx methods achieve consistent performance over short periods, such as a single day, and if they remain stable over longer durations, spanning years, notwithstanding potential subject variations during such intervals.

This paper aims to investigate the aforementioned points by assessing the reproducibility and variability of three excitation methods for 3D nonselective imaging: default (vendor supplied) RF excitation, subject‐tailored 4kT‐points pTx pulses (TPs), and calibration‐free 4kT‐points universal pulses (UPs). To achieve this objective, we conducted, to our knowledge, the first longitudinal study of its kind, involving three different MRI operators who performed rescans at 1‐year and 2‐year intervals, with interday scans performed by two different operators.

## 2 METHODS

### 2.1 MR scanner and hardware

MRI acquisitions were conducted using a whole‐body investigational 7 T MRI scanner (Magnetom 7 T, Siemens Healthineers, Erlangen, Germany) equipped with a 32‐element pTx‐capable RF body array operating in 8Tx/32Rx mode (MRI.TOOLS, Berlin, Germany). Each Tx channel uses four fixed‐wired coil elements for RF transmission, whereas all 32 coil elements are used individually for RF signal reception (Rx). The coil used in this study was identical to the one used for in vivo measurements in previous investigations, and strict adherence to safety limits was ensured, with execution following the established protocols from those earlier studies.^22^, ^25^, ^26^, ^28^

The reconstruction of $B_{1}^{+}$ maps, manual slice‐by‐slice selection of 3D heart regions of interest (ROIs), RF pulse design, and the development of pulse files were executed on a separated workstation equipped with 12 cores operating at 2.1 GHz and 128 GB of RAM.

### 2.2 Volunteer information

The $B_{1}^{+}$ library group, denoted henceforth as the “Library” group, from which the UP was designed were taken from a previous study (15 males and 7 females, aged 21–66 years, with body mass indexes [BMIs] ranging from 19.8 to 28.3 kg/m^2^).^28^ In this work, additional MRI scans were performed on 6 healthy subjects not included in the aforementioned Library group following approval from an institutional review board and written informed consent (3 males and 3 females, aged 25–33 years, with BMIs ranging from 19.5 to 35.3 kg/m^2^). The 6 subjects were scanned four times and grouped accordingly: (i) first scan (Scan 1), performed between 2020 and 2021; (ii) rescans after a year (Scan 2); (iii) rescans after 2 years (Scan 3); and (iv) intra‐day rescans of Scan 3 group (Scan 4).

### 2.3 Volunteer positioning

Three different MRI operators were involved in this study. Each operator received identical instructions, outlined as follows: At the onset of each scan, the posterior section of the coil should be centered to the subject's heart along the head–foot axis, whereas the anterior section should be adjusted using anatomical landmarks and subject feedback to maintain a 2‐cm separation between the coil and the subject's chin. The isocenter should be located 5 cm away from the center of the anterior section in the direction of the head. These instructions are consistent with those applied in previous studies.^22^, ^25^, ^26^, ^28^

### 2.4 B_1_ ^+^ mapping

For each subject, relative 3D $B_{1}^{+}$ maps of the chest were obtained using a radial phase‐encoded (RPE) trajectory while maintaining shallow breathing (nominal FA = 20°, echo time/repetition time = 2.02/40 ms, field of view = [250 × 312 × 312] mm^3^, resolution = [3.9 × 3.9 × 2] mm^3^, 256 RPE lines with golden‐angle increments,^33^, ^34^ acquisition time [TA] = 3 min 25 s). After the scan, the raw data were transferred to a remote workstation and reconstructed during the subsequent MR scan. Despite acquisition during respiration, the reconstructed 3D $B_{1}^{+}$ maps were free from visible motion‐related artifacts. A slice‐by‐slice manual delineation of a ROI over the entire heart was performed on the sum‐of‐magnitudes of the $B_{1}^{+}$ maps for each subject. This ROI served as a binary mask for designing dynamic RF pulses. To ensure comparable regularization terms and nominal FAs across the subjects, the relative 3D $B_{1}^{+}$ maps were normalized based on the mean value of the sum of magnitudes in the subject's heart ROI.

### 2.5 RF pulse design

Three different pTx settings were investigated in this work: (i) default (phase‐only RF shim set by the coil manufacturer so that sufficient $B_{1}^{+}$ is delivered across the heart and the aorta), (ii) TP (subject‐tailored 4kT‐points pulses), and (iii) UP (universal 4kT‐points pulses designed from all 22 $B_{1}^{+}$ maps in the Library group).^28^ All RF pulses and gradient blips were designed using the aforementioned relative 3D $B_{1}^{+}$ maps in *MATLAB* (The MathWorks, Natick, MA, USA), using an interleaved greedy and local optimization algorithm^21^, ^35^ to iteratively determine the optimal k‐space location of each kT‐point.^22^, ^25^

### 2.6 RF pulse evaluation

**FIGURE 1:**
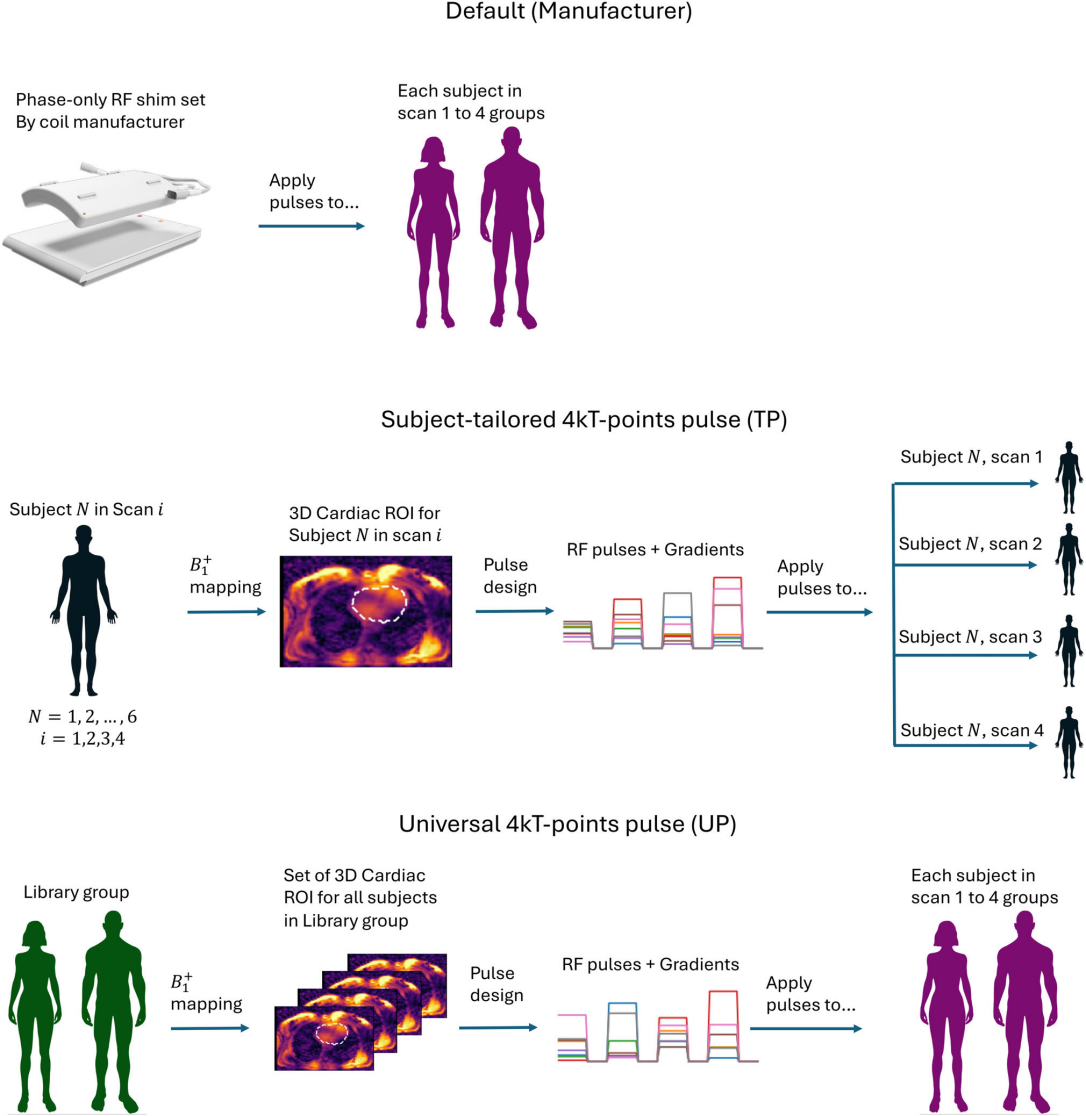
Study overview. To investigate the performance and reproducibility of different parallel‐transmission settings, radiofrequency (RF) pulses and gradient blips were designed and applied in various configurations. Tailored kT‐points pulses (TPs) were tailored to each subject and scanned individually and evaluated in all four scans of the subject for whom they were designed. In contrast, the universal kT‐points pulse (UP) was applied to all subjects and scans. 3D, three‐dimensional; ROI, region of interest.

To evaluate the performance and reproducibility of the different pTx RF pulses, we used data sets from four separate scan groups (Scans 1 to 4). In a first step, TPs were calculated for each subject in each scan group. These TPs were then grouped based on the scan they were optimized for: Config. 1 for Scan 1 TPs, Config. 2 for Scan 2 TPs, and so on. Next, TPs from each configuration (Config. 1 to 4) were applied to all four scan groups. The default RF shim pTx setting and the UP were applied to all subjects within each scan group without any further modification. The pulse's performance was evaluated in all subjects, assessing the coefficient of variation (CV) of the simulated FAs within the ROI. Figure 1 illustrates this process, depicting the application of default, TP, and UP to each subject.

### 2.7 Experimental validation

Isotropic, 3D gradient‐echo (GRE) images were acquired with TP and UP to qualitatively validate the simulated FA predictions. RPE trajectory–based 3D GRE scans were used (nominal FA = 10°, echo time/repetition time = 1.75/3.7 ms, field of view = 250 × 312 × 312 mm^3^, 1.4 × 1.4 × 1.4 mm^3^ resolution, 256 golden‐angle spaced RPE lines, TA = 3 min 35 s) as described in more detail in previous work.^28^

The results obtained from applying UP and TPs in the four scan groups were later categorized into three distinct studies: (i) same day acquired by different operators (Scans 3 and 4), (ii) 1‐year time interval acquired by the same operator (Scans 1 and 3); and (iii) 2‐year time interval acquired by different operators (Scans 1, 2 and 4). In the same day acquired by different operators' study, results were obtained by applying TPs in Config. 3 to Scan 3 and Config. 4 to Scan 4 and UP to the Scan 3 and Scan 4 groups. For the 1‐year time interval acquired by the same operator study, results were produced by applying TPs in Config. 1 to Scan 1 and Config. 3 to Scan 3 and UP over the Scan 1 and Scan 3 groups. Finally, for the 2‐year time interval acquired by different operators' study, results were generated by applying TPs in Config. 1, Config. 2, and Config. 4 and UP over the Scan 1, Scan 2, and Scan 4 groups, respectively.

### 2.8 Statistical analysis

The CV values obtained from the FA predictions for each of the three investigations considered in this work were used in a nonparametric Wilcoxon signed‐rank test, performed in *Python* (Python Software Foundation, Delaware, USA) to evaluate statistical differences. Statistical significance was determined at varying *p*‐values below 0.05, depending on the number of data‐set comparisons, due to Bonferroni correction. Significant outcomes were denoted as “S,” while nonsignificant results were labeled as “NS” in the figures.

To gain deeper insights into the behavior of CV prediction obtained for the different shim methods over different timeframes, three key parameters were quantified. These parameters are defined as follows:

1. Inertia ($I_{\mathrm{CV}}$): Inertia serves as a measure of the spread or dispersion of CV predictions. It helps us understand how data points are distributed and whether they cluster tightly or spread out over the CV data space. A small $I_{\mathrm{CV}}$ indicates more precise CVs across different scan groups. Inertia is defined as

(1) $$I_{\mathrm{CV}}=\sqrt{\frac{1}{V\cdot S}\sum_{v}\;\sum_{s}{\left(CV_{v,s}-{\bar{x}}_{s}\right)}^{2}},$$

where $CV_{v,s}$ is the CV value for the v th subject in the s th scan group; ${\bar{x}}_{s}$ is the position of the centroid of the cluster of CV data points for the s‐th scan group; V is the number of subjects; and S is the number of scans performed to each subject, v={1,2,…,V}, and s={1,2,…,S}.

2 Distance from the origin of the CV space to centroid ($|\bar{x}|$): This parameter quantifies the overall location of the centroid of the CV data cluster with respect to the origin of the CV data space. It provides information about the magnitude of the CV values for each shim method. Mathematically, it is defined by

(2) $$\left|\bar{x}\right|=\sqrt{\sum_{s}{\bar{x}}_{s}^{2}}.$$

3 Perpendicular distance from the centroid to the identity line ($d_{⊥}$): The $d_{⊥}$ parameter is a crucial indicator of how well the CV predictions align with the identity line in the CV data space. It helps in assessing the correlation or similarity of the data points for different scan groups. The smaller $d_{⊥}$, the more correlated are the data sets from which this parameter was obtained. This quantity is given by

(3) $$d_{⊥}=\sqrt{\sum_{s}{\left(\frac{1}{|s^{\prime }|}\sum_{s\prime }{\bar{x}}_{s\prime }-{\bar{x}}_{s}\right)}^{2}},$$

where $s^{\prime }=s=\left\{1,2,\ldots ,S\right\}$ and $|s^{\prime }|$ is the cardinality of the set $s^{\prime }$.

The parameters $I_{\mathrm{CV}},|\bar{x}|$, and $d_{⊥}$ were defined in such a manner that small values of these parameters, when obtained for a particular shim method, indicate that said method achieve reproducible results while minimizing data dispersion and CV values.

## 3 RESULTS

**FIGURE 2:**
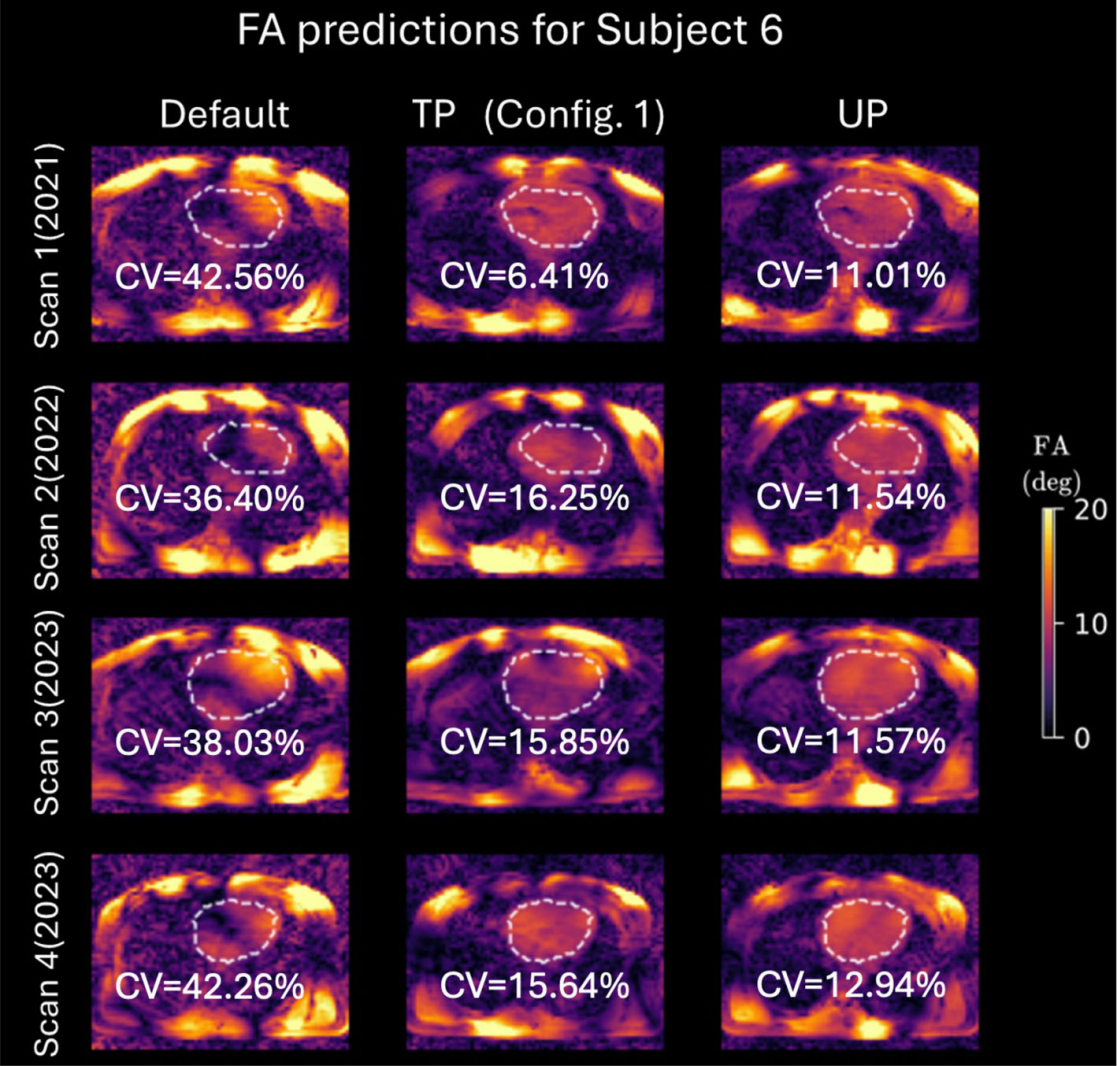
Flip‐angle (FA) predictions for the human heart using the three parallel‐transmission settings: default, tailored kT‐points pulse (TP; Conf. 1), and universal kT‐points pulse (UP). The white‐dashed lines delineate the borders of each cardiac region of interest. The data represent all four scans performed on Subject 6. Notably, the TP method, specifically optimized for Scan 1, demonstrates superior performance compared with UP only in Scan 1. In the remaining scans, UP exhibits better results. CV, coefficient of variation.

Figure 2 shows FA predictions for a representative transversal two‐dimensional slice (from a 3D volume) centered on the heart, which is outlined by a dashed line. Depicted are the results using the default RF shim, a TP tailored on Scan 1, and the calibration‐free UP for a single subject (Subject 6) across all four scans. Qualitatively, both TP and UP exhibit significant improvements in FA homogeneity within the cardiac region compared with the default RF shim, with no observable FA dropouts. Quantitatively, the calibration‐free UP consistently outperforms the default RF shim across all scans, achieving CV values within a narrow range of 11% to 12.9% when evaluated in the 3D heart ROI. Compared with UP, the TP demonstrates a 2‐fold reduction in CV (6.4%) for the scan it was specifically designed for (Scan 1). However, its performance degrades when applied to other scans of the same subject it was not tailored for, leading to a CV increase of approximately 40% compared with UP.

**FIGURE 3:**
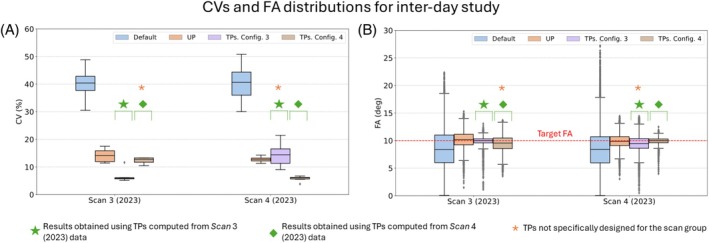
(A) Coefficient of variation (CV) of the flip angles (FAs) in the three‐dimensional heart region of interest obtained from default, tailored kT‐points pulse (TPs), and universal kT‐points pulse (UP) across interday scan groups acquired by two different MR operators. Results marked with a green star represent the outcomes obtained by applying TPs designed from Scan 3 data, whereas results marked with a green diamond denote the results obtained by applying TPs designed from the Scan 4 data. The orange asterisk indicates the results obtained by applying a TP specifically not designed for the corresponding scan group. (B) The corresponding FA distributions.

Figure 3 depicts CV predictions for all subjects in interday scan groups acquired by two different MR operators. According to the results reported in Figure S1A, predicted CV values obtained for TPs and UP in each scan group and TPs configuration are significantly different from the results obtained for the default shim method. On the other hand, CV values from the UP method do not statistically differ from TPs predictions obtained by applying a tailored pulse specifically not designed for the corresponding scan group (hereafter referred to as “nTSGs”), with median values ranging between 10% and 20%. However, for scan groups for which tailored pulses were designed (henceforth denoted as “TSGs”), CV values for the TPs method reach approximately 5% with notably minimal dispersion, rendering these results significantly distinct from both other TP results and UP results. FA distributions in Figure 3B also suggest that UP predictions for all Scan groups, as well as TP predictions for TSGs, reach a median FA value equal to the target FA of 10°. However, TP predictions for nTSGs reach median values lower than the target FA, with a higher dispersion.

**FIGURE 4:**
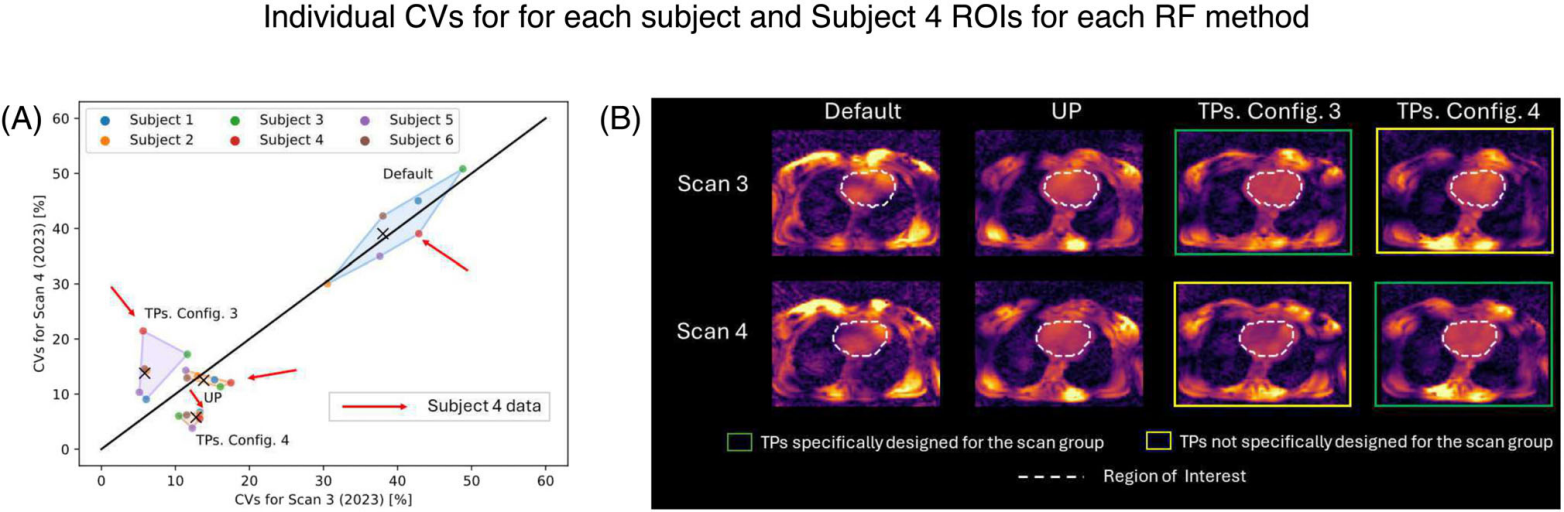
(A) Coefficient of variation (CV) distribution for each subject and each shim method considering data from Scan 3 and Scan 4 groups. The colored areas are determined by the convex hull formed by the points representing the CV values for each subject, considering a fixed shim method. The median of each cluster of points is marked by a black X. The red arrows point to the datapoints of Subject 4. (B) A set of transverse slices obtained from flip‐angle (FA) predictions for Subject 4, considering each scan group and each shim method. FA predictions in Subject 4 obtained using tailored kT‐points pulses (TPs) specifically designed for the subject and the corresponding scan group are framed by a green rectangle, whereas FA predictions obtained using TPs specifically designed for the subject but not for the corresponding scan group are framed by a yellow rectangle. The mask used for pulse design is depicted as a white dashed line. RF, radiofrequency; ROI, region of interest.

Figure 4 illustrates individual CV data for each subject in interday scan groups, which were presented in a summarized form in Figure 3. On the one hand, the dispersion of the cluster of points corresponding to TP Config. 3 is notably larger compared with the dispersion of the other clusters of points, whose CV values lie below 15%. This disparity arises from the CV values of Subjects 3 (18%, 11%) and 4 (21%, 5%) obtained from Scan 3 and Scan 4 data, respectively, indicating that this specific TP configuration does not maintain the accuracy of CV values for an interday study. It is noteworthy that the consistency of CV values for both TP Config. 3 and TP Config. 4 is questionable, as their corresponding cluster median values lie relatively far from the identity line. This discrepancy can be quantitatively observed in Figure S1B, left, where the value of $d_{⊥}$ for both TP configurations is between 4 and 5 times larger compared with the other methods. On the other hand, the UP cluster of points exhibits the smallest dispersion ($I_{\mathrm{CV}}˜14\%$) and distance to the identity line ($d_{⊥}˜1\%$) simultaneously, suggesting that the UP method maintains both precision and accuracy in terms of CV values for an interday study, demonstrated by the values of $I_{\mathrm{CV}}$ and |x| below 20 in Figure S1B (left). The reason for the high dispersion of the TP Config. 3 cluster of points can be qualitatively observed in Figure 4B in Subject 4. Although applying TPs specifically designed for this subject and for the Scan 3 group to the same subject in the corresponding scan group achieves FA homogenization, intensity dropouts occur when the same pulses are applied to the same subject but in the Scan 4 group. This phenomenon results in more dispersed and higher CV values, observed by the value of $I_{\mathrm{CV}}$ above 20% for the nTSG in Figure S1B (left).

**FIGURE 5:**
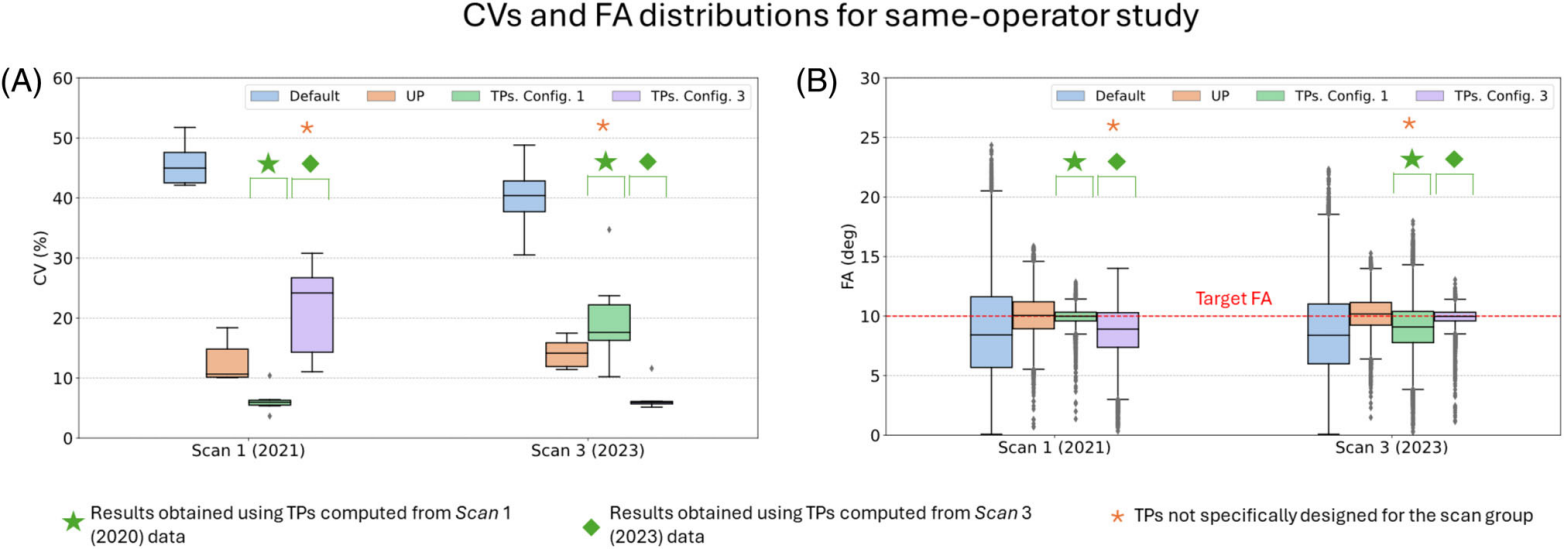
(A) Coefficient of variation (CV) predictions obtained from default, tailored kT‐points pulse (TP), and universal kT‐points pulse (UP) shim methods across scans conducted by the same operator. Results marked with a green star represent the outcomes obtained by applying TPs designed from the Scan 1 data, whereas results marked with a green diamond denote the results obtained by applying TPs designed from the Scan 3 data. The yellow asterisk indicates the results obtained by applying a tailored pulse specifically not designed for the corresponding scan group. (B) The corresponding flip‐angle (FA) distributions.

Figure 5 illustrates the CV predictions for all subjects in scans performed by the same operator but performed within a 2‐year timespan. Similar to the interday findings, predictions from the default RF shim significantly differ from those of the TPs and UPs with CV median values greater than 40%. Moreover, UP results exhibit statistical disparities from all TPs results according to Figure S1A. TP predictions for TSGs demonstrate median CV values of approximately 5% with minimal dispersion (from approximately 0.5% to 0.8%), whereas TP predictions for nTSGs yield median values of 18% and 25% with comparatively higher dispersion for Config. 1 in Scan 3 and Config. 3 in Scan 1, respectively. UP results display CV values ranging from 11% to 14% with a moderate dispersion (˜3.5% to ˜5%), falling between the extremes of TP results. Furthermore, FA distributions in Figure 5B suggest that UP predictions for all Scan groups, as well as TP predictions for TSGs, achieve a median value equal to the target FA of 10°. However, TP predictions for nTSGs attain median values lower than the target FA, with a higher dispersion.

**FIGURE 6:**
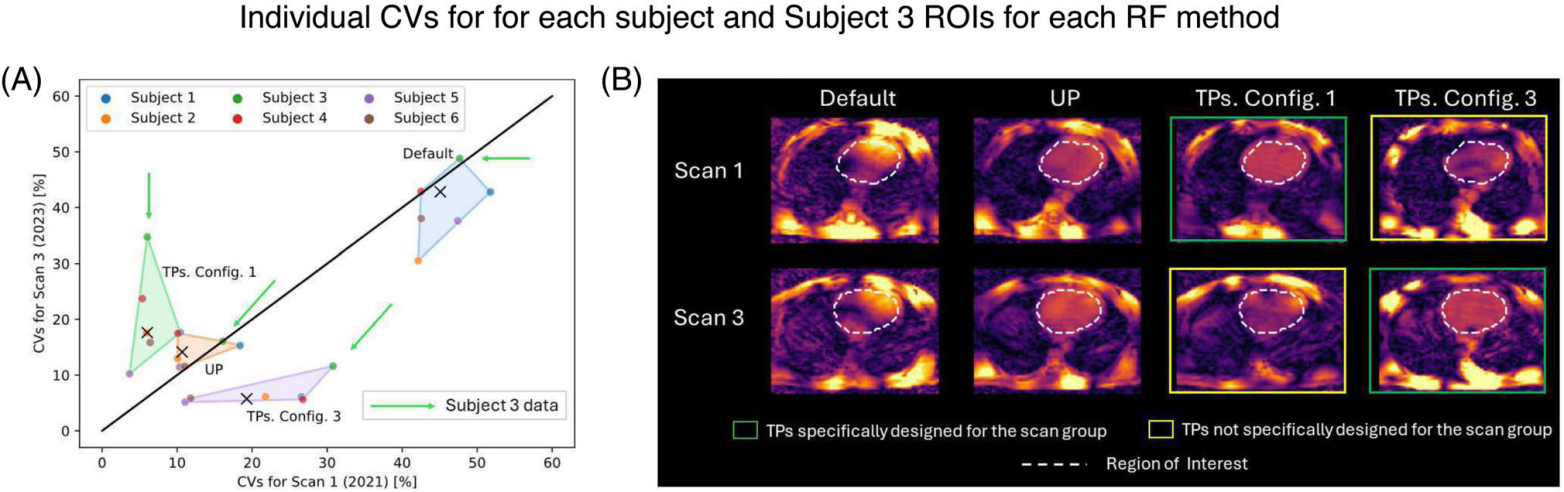
(A) Coefficient of variation (CV) distribution for each subject and each shim method considering data from the Scan 1 and Scan 3 groups. The colored areas are determined by the convex hull formed by the points representing the CV values for each subject, considering a fixed shim method. The median of each cluster of points is marked by a black X. The green arrows point to the datapoints of Subject 4. (B) A set of transverse slices obtained from flip‐angle (FA) predictions for Subject 3, considering each scan group and each shim method. FA predictions in Subject 3 obtained using tailored kT‐points pulses (TPs) specifically designed for the subject and the corresponding scan group are framed by a green rectangle, whereas FA predictions obtained using TPs specifically designed for the subject but not for the corresponding scan group are framed by a yellow rectangle. The mask used for pulse design is depicted as a white dashed line.

Figure 6 presents individual CV data for each subject in same‐operator scan groups, which were presented in a summarized form in Figure 5. Similar to the interday results, presented in Figure 4, TPs exhibit the largest dispersion and lower precision compared with results obtained by applying UP, observed by the $I_{\mathrm{CV}}$ values >30% and $\left|\bar{x}\right|$ values >20% for TP data in Figure S1B (center). However, the dispersion in TP results has increased at least 40% compared with the interday study, as confirmed by the values of $I_{\mathrm{CV}}$ in Figure S1B (center). This dispersion in TP results primarily stems from median CV values obtained from Subjects 3, 4, and 5. A closer examination of Subject 3's FA predictions in Figure 6B confirms that applying TPs for a scan group for which they were not designed can introduce noticeable intensity dropouts in the cardiac region. This dropout is responsible for the relatively high dispersion in TP CV data (see images marked by a yellow rectangle in Figure 6B). Nevertheless, UP results maintain consistency in FA predictions across different scan groups.

**FIGURE 7:**
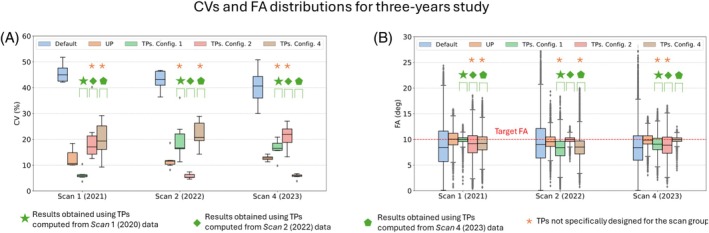
(A) Coefficient of variation (CV) predictions derived from default, tailored kT‐points pulse (TP), and universal kT‐points pulse (UP) shim methods across interyear scan groups, performed by various operators. Results marked with a green star represent the outcomes obtained by applying TPs designed from Scan 1 data, whereas results marked with a green diamond and a green pentagon denote the results obtained by applying TPs designed from the Scan 2 and Scan 4 data, respectively. The yellow asterisk indicates the results obtained by applying a tailored pulse specifically not designed for the corresponding scan group. (B) The corresponding flip‐angle (FA) distributions.

Figure 7 depicts the CV predictions for inter‐year scan groups acquired by three different MR operators (Scans 1, 2, and 4). Consistent with same‐operator and inter‐year studies, median CV values using the default RF shim significantly differ from those of the TP and UP methods by more than 20%. Moreover, TP results exhibit significant differences from UP results in all cases, as illustrated in Figure S1A. Median CV values for TP and UP predictions fall within similar ranges (approximately 5% for TP and 11% for UP), as observed in inter‐day and same‐operator studies. Furthermore, FA distributions in Figure 7B suggest that UP predictions, along with TP predictions for TSGs, are the only ones achieving a FA median value equal to the target FA of 10°, consistent with inter‐year and same‐operator studies.

**FIGURE 8:**
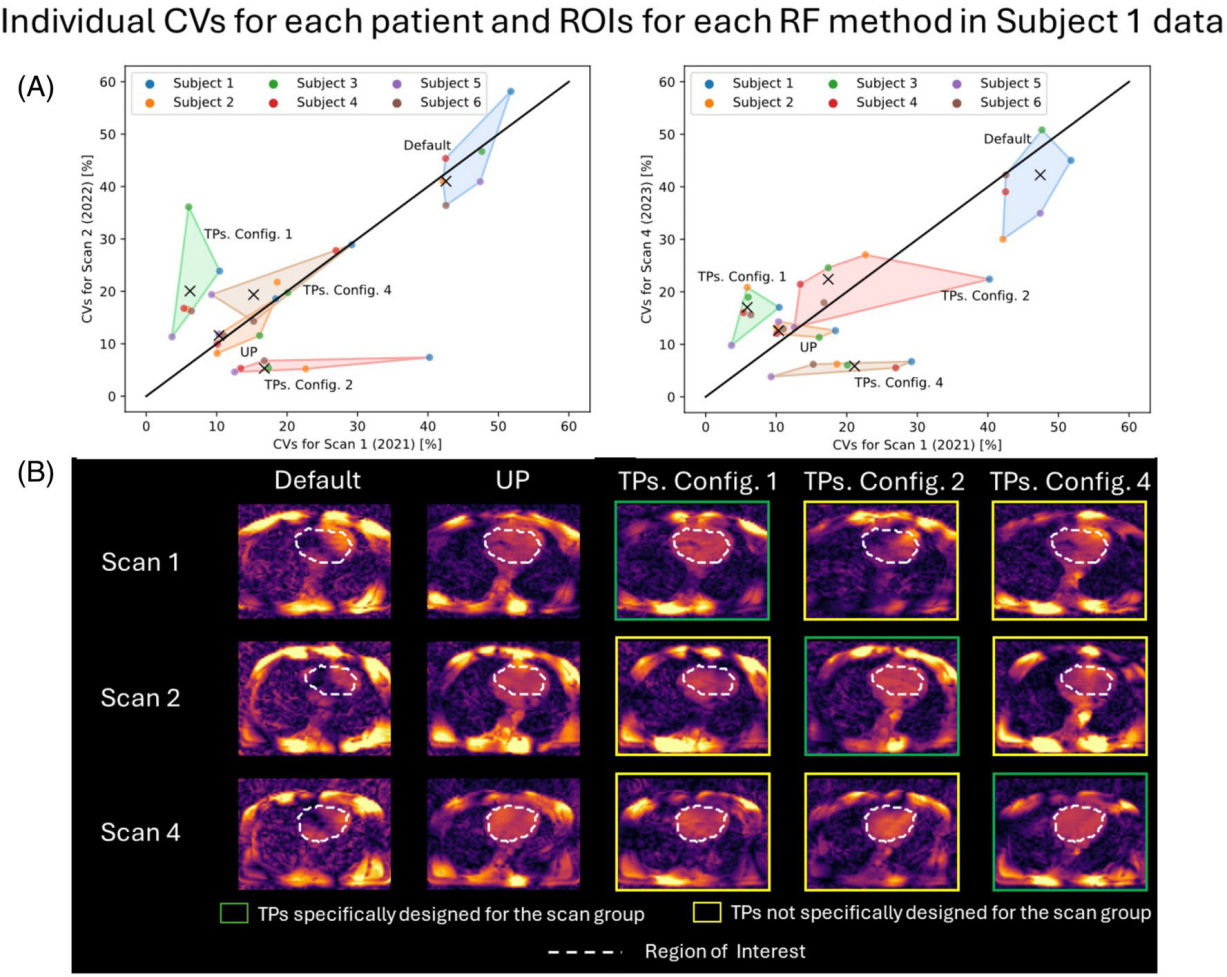
(A) Two‐dimensional projections of the coefficient of variation (CV) distribution for each subject and each shim method considering data from the Scan 1, Scan 2, and Scan 4 groups. The colored areas are determined by the convex hull formed by the points representing the CV values for each subject, considering a fixed shim method. The median of each cluster of points is marked by a black X. (B) A set of transverse slices obtained from flip‐angle (FA) predictions for Subject 1, considering each scan group and each shim method. FA predictions in Subject 1 obtained using tailored kT‐points pulses (TPs) specifically designed for the subject and the corresponding scan group are framed by a green rectangle, whereas FA predictions obtained using TPs specifically designed for the subject but not for the corresponding scan group are framed by a yellow rectangle. The mask used for pulse design is depicted as a white dashed line. RF, radiofrequency; ROI, region of interest; UP, universal kT‐points pulse.

Figure 8 illustrates individual CV data for each subject in 3‐year scan groups, which are presented in a summarized form in Figure 5. Consistent with the inter‐day and same‐operator results, TPs demonstrate the largest dispersion and less precision compared with results obtained by applying UP, observed by the values of $I_{\mathrm{CV}}$ above 40% and the values of $\bar{|x}|$ above 30% in Figure S1B (right). However, the dispersion in TP results has further increased compared with the same‐operator study, as evidenced by the values of $I_{\mathrm{CV}}$ in Figure S1B. This dispersion in TP results primarily arises from median CV values obtained from Subjects 1, 4, and 5, which are the ones that determine the convex hull of the cluster of CV points. A closer look at Subject 1's FA predictions in Figure 8B confirms that applying TPs for a scan group for which they were not designed introduces noticeable intensity dropouts in the cardiac region. This dropout accounts for the relatively high dispersion in TP CV data (see images marked by a yellow rectangle in Figure 8B). Nonetheless, UP results maintain consistency in FA predictions across different scan groups, consistent with the same‐operator and interday studies.

**FIGURE 9:**
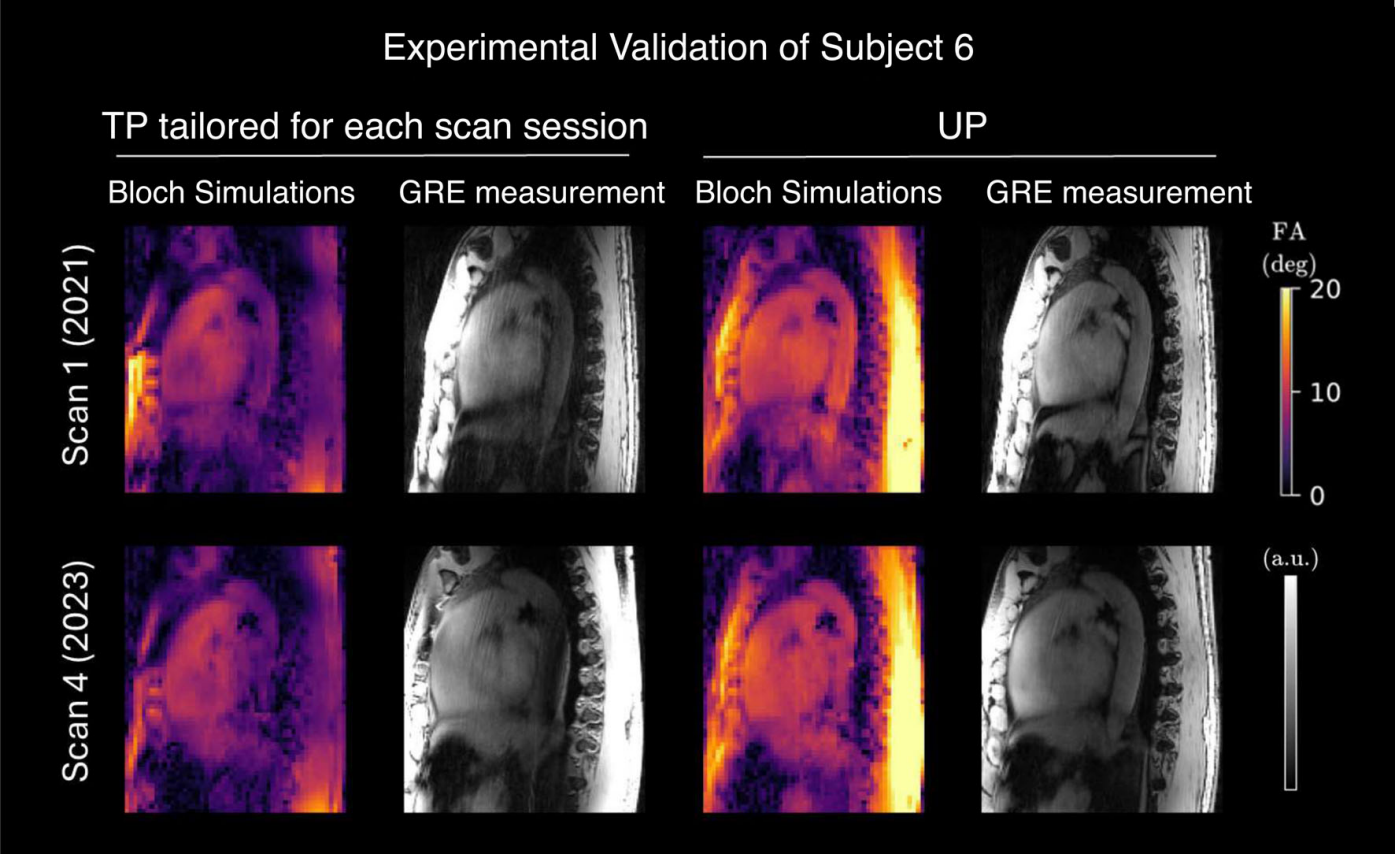
Bloch simulations for a single sagittal slice within a three‐dimensional cardiac volume using appropriately designed tailored kT‐points pulses (TPs) for Scan 1 and Scan 4 of Subject 6 and the universal kT‐points pulse (UP), designed to homogenize the human heart region in the $B_{1}^{+}$ library. The remaining signal variations observed in the anterior–posterior (AP) direction are likely attributed to received ($B_{1}^{-}$) variations. FA, flip angle; GRE, gradient echo.

Figure 9 presents a representative sagittal slice of 3D Bloch simulations and 3D GRE images acquired using two TPs tailored for each scan session of Subject 6 and using the precomputed UP. A qualitative correspondence between the FA prediction and GRE images was observed. Remaining discrepancies in signal intensity are likely attributed to receive profile variations. These results demonstrate the feasibility of UP and appropriately designed TPs for acquiring the human heart over a time span of 3 years at UHF.

## 4 DISCUSSION

This paper aims to investigate the reproducibility and variability of three RF pTx excitation strategies: vendor‐supplied default RF shim, subject‐tailored TPs, and calibration‐free UP. To achieve this, rescans were conducted by different MRI operators at a 1‐year and at a 2‐year interval, with additional interday scans. This work, to our knowledge, is the first longitudinal study of its kind, quantifying the consistency of real‐world operator performance across multiple subjects over a 3‐year period.

The precomputed UP, designed for a library of 22 $B_{1}^{+}$ maps, proved to be the pTx excitation strategy that provides the most reproducible results for interyear, interday, and same‐operator studies compared with the TPs, while minimizing CV data dispersion and median CVs. Even though CV values obtained from TP results for TSGs were consistently lower and less dispersed than those from UP results, these reductions were not sustained over interday and interyear periods, where the position or anatomy of the subjects may have changed. Furthermore, TP results for nTSGs fell short of achieving a median target FA equal to the target FA in interday, interyear, and same‐operator studies, positioning UP as the optimal method for achieving the target FA while maximizing FA uniformity. Qualitatively, while UP results show similarity to those acquired with TPs, offering the benefit of uniform regions extending to the aorta, there is an observable inclination for FA overestimation in the posterior region adjacent to the spinal column, close to one of the coil elements. In summary, while subject‐tailored TPs exhibited nearly twice the CV improvement over precomputed UPs across all subjects, their reproducibility was lower. Consequently, they should be computed on a per‐subject and per‐scan basis, even when the same subject is acquired in an intraday study.

This research was initially motivated by findings from a prior study,^36^ which revealed inconsistencies in CV values and FA distributions across different pTx excitation strategies, notably default RF shim and TPs, when comparing scans taken with a 1‐year interval for the same subject. In contrast, UPs demonstrated resilience against FA variations, resulting in consistently reproducible CV values. However, drawing definitive conclusions regarding the long‐term robustness of each shim method based solely on two scan sessions with a 1‐year time resolution proved insufficient. To enhance the reliability of our findings, two additional scan sessions (Scan 3 and Scan 4 sessions) were incorporated into this study, extending the observation period up to 3 years and including a same‐day study. Scan 3 was conducted by the same operator as Scan 1, whereas Scan 4 was performed by a new operator. This approach allowed for direct comparisons between multiple MRI operators—a factor not explicitly considered in Aigner et al.^36^

When comparing the results from the same‐operator study with those from the 3‐year study, which both encompass data from 2021 and 2023, it becomes apparent that MRI operators may influence the outcomes. In instances where the same MRI operator conducts two scans in 2 different years (refer to Figure 6A), the dispersion of the cluster of points in the CV space for all shimming methods decreases compared with when two different MRI operators carry out each scan session separately (refer to Figure 8A, right). This suggests that when the same operator conducts scans in 2 different years, CV values will be more precise compared with the results obtained when two different MRI operators perform the scans. However, considering only two scan sessions may not be sufficient to make a definitive statement regarding the effect of the MRI operator on the measurements.

Other aspects not explicitly assessed in Aigner et al.^36^ are the limitations of the study related to the used body coil. The first limitation is the effect of coil placement on the results produced by each shim method. The robustness of UP against variations in coil placement was observed by Gras et al.^29^ and Le Ster et al.^37^ for brain imaging at 7 T, as well as by Aigner et al.^28^ for cardiac imaging. However, robustness over short‐term and long‐term timeframes was not explicitly examined. The observations regarding CV and FA distributions across the interyear, same‐operator, and 3‐year studies suggest that the two methods that we can assure are resilient against variation in coil placement are UPs and TPs applied to TSG, as CV values and FA distributions were consistent across the three different studies considered in this work, disregarding factors such as the MRI operator or the period of time between scan sessions. This is also confirmed by the experimental validation shown in Figure 9 for UP results.

This investigation extended beyond FA homogeneity to encompass RF voltage and power requirements. Consistent with prior observations,^25^, ^28^ both TP and UP designs exhibited an approximate 3‐fold increase in mean peak voltage and a 2‐fold increase in average RF power compared with the default RF shim pulse. Please note that these values strongly depend on the chosen regularization factor. While TPs and UPs demonstrated comparable peak RF voltage and power demands, they resulted in different complex RF weights and 3D gradient blip characteristics. Furthermore, a nonuniform interchannel RF power distribution was observed for both TPs and UPs, similar to previous works.^25^, ^28^

The UP was designed for a library of 22 $B_{1}^{+}$ maps acquired from subjects exhibiting substantial anatomical variations. This raises the question of whether UPs tailored to specific subpopulations could offer advantages in terms of FA homogeneity and/or RF power requirements. Previous work^28^ demonstrated that group‐specific UPs (e.g., for female subjects or those with high BMI) yield improved FA homogeneity and reduced RF power demands, with improvements on the order of 20% compared with a general UP, consistent with findings reported in brain studies.^38^

Recent advancements in tailored pTx pulse design have focused among others on reducing lengthy adjustment times and broadening applicability. These efforts include strategies to shorten $B_{1}^{+}$ mapping duration, such as artificial intelligence–based estimation of $B_{1}^{+}$ maps from localizer data,^39^ accelerated non‐Cartesian $B_{1}^{+}$ mapping,^40^ rapid (one breath hold) $B_{1}^{+}$ mapping,^41^ efficient implementations to accelerate pulse design computations,^42^ or online per‐subject pulse calculation to minimize adjustment times in brain imaging.^43^ Combined with automated target volume segmentation, such approaches could substantially reduce the reported 10 min adjustment time and enable rapid tailored pTx, even outside of the brain. Alternative pTx approaches involve hybrid strategies combining TP and UP design methods to leverage the advantages of both, while minimizing adjustment overhead. These intermediate strategies can be implemented through tailored adjustments of UPs using rapid calibration scans to achieve subminute adjustment times^27^, ^36^ or machine learning–based SmartPulses, where localizer information is used to select optimal precomputed pulses based on subject‐specific labels.^44^

A limitation of this work is the lack of a rigorous experimental validation for the default RF shim configuration and the simulated results. However, extensive prior research^25^, ^26^ provides experimental confidence in the default RF shim and the performed Bloch simulations. Although the coil used in this study provides a large excitation field, its maximum $B_{1}^{+}$ efficiency at the body center is limited to approximately 2.4 μT/√kW. This restricts the achievable FA to approximately 10° without increasing RF pulse durations.^28^ However, an increased number of Tx channels (e.g., 16 instead of 8) and more powerful amplifiers (e.g., 2 kW instead of 1 kW per channel), as recently demonstrated at 10.5 T,^45^ offers great potential for fully realizing the capabilities of pTx pulses. This is particularly true for large‐FA pulses, which are crucial for generating the improved contrast necessary for clinical cardiac imaging, especially for 3D applications, including inversion or saturation pulses to enable high‐resolution T_1_‐weighted sequences or T_1_ mapping.

To avoid the additional scan time for absolute 3D $B_{1}^{+}$ mapping,^26^ we opted to investigate only relative $B_{1}^{+}$ maps for this longitudinal study. This limits the analysis to small FAs, which is in accordance with the RF coil limitations. Without knowledge of the absolute $B_{1}^{+}$, however, variations in the actual FA achieved during the experimental application might occur. This limitation could be addressed by using an absolute $B_{1}^{+}$ map in a single slice, or an AI‐based absolute $B_{1}^{+}$ mapping method, which would offer a fast option to scale the relative maps to absolute $B_{1}^{+}$ maps.

Another limitation of this work is the small number of subjects per scan session. Even though the number of subjects in each scan session was sufficient to show how factors such as MRI operators and coil position affect the use of TPs and UPs over short and long periods of time, including a larger number of subjects with a wide variety of BMI and age ranges would further improve confidence in the results. Additionally, a larger pool of subjects could help obtain sufficient statistics to study the impact of intersubject variations in detail.

Regarding subject positioning, it is evident from Figure S1B that, regardless of the period of time or the operator, UP consistently provides the lowest values of $I_{\mathrm{CV}},\left|\bar{x}\right|$, and $d_{⊥}$ simultaneously, indicating that this method is also robust against variations in subject positioning. In contrast, the results for TPs vary depending on the time period considered or the operator, making it unclear whether the differences in CV distributions obtained using TPs are due to variations in subject positioning or other factors, such as changes in the scanner over time, intersubject variation, or other potential reasons.

Considering the previous discussion, UPs have proven to be the most robust method, providing consistent results over time, even when different operators are involved. For interday studies, TPs also demonstrated to be a good option, as this method yields the lowest CV values compared with the default and UP methods. However, TPs require offline calibration time, which the precomputed UPs do not. For long‐term studies, TP results are inconsistent, as indicated by the relatively high $I_{\mathrm{CV}}$ values in Figure S1B (right). Additionally, the need for offline calibration time in each scan session puts TPs at a disadvantage compared with UPs. However, this disadvantage can be reduced by applying methods that combine the advantages of TP and UP methods to enable rapid pTx.^27^, ^37^, ^44^

## 5 CONCLUSION

The suitability of UPs and TPs for 7T cardiac FA homogenization is confirmed, with UPs demonstrating consistency in managing FA variations across different subjects and coil placements in 3D body imaging at 7 T. However, TPs designed for a specific scan session and patient are not optimal for other sessions, highlighting the need for universal solutions when robustness is required. The results suggest using UPs or TPs designed specifically for a scan group rather than reusing pulses from previous sessions. For FA consistency, UPs should be the method of choice, whereas TPs are recommended when minimizing CV values is the priority.

## Supporting information


**FIGURE S1.** (A) Statistical significances obtained from the coefficient of variation (CV) results obtained from different shim methods, scan groups, and tailored kT‐points pulse (TP) configurations. A significant difference between two scan groups is denoted by a white “S” in a green background, while nonsignificant differences are indicated by a white “NS” in a red background. The prefixes “D,” “TP,” and “UP” denote results from default, TP, and universal kT‐points pulse (UP) shim methods, respectively. Additionally, identifiers c1 to c4 correspond to results from TPs in Config.1 to Config.4, whereas suffixes s1 to s4 denote results from Scan 1 to Scan 4 groups, respectively. (B) Values of $I_{\mathrm{CV}},|\bar{x}|$, and $d_{⊥}$ for each shim method, normalized to the maximum value reported among all shim groups. Top‐left and bottom‐left figures correspond to data obtained from the Scan 3 and Scan 4 data sets. The top‐center and bottom‐center figures correspond to data obtained from the Scan 1 and Scan 3 data sets. Top‐right and bottom‐right figures correspond to data obtained from the Scan 1, Scan 2, and Scan 4 data sets.
**Figure S2.** Three‐dimensional flip‐angle predictions for the human heart using default, tailored‐pulse (TP; Conf. 1) and universal kT‐points pulse (UP) shim methods. The data correspond to all four scans conducted on the sixth subject.
**Figure S3.** Pulse diagram of complex radiofrequency (RF) voltages (magnitude and phase) and three‐dimensional (3D) gradient blips of the universal kT‐points pulse (UP) and tailored kT‐points pulse (TP) method (all configurations) of the sixth subject. The RF voltage of each parallel‐transmit (pTx) pulse was scaled to achieve a nominal flip angle (FA) of 10° in the three‐dimensional (3D) heart region of interest (ROI), allowing for quantitative comparisons of RF voltages between different pulses.

## Data Availability

The design code and example $B_{1}^{+}$ maps are available at https://github.com/chaigner/UP_body.
