# Supplementary material for: Reproducibility of tailored and universal nonselective excitation pulses at 7 T for human cardiac MRI: A 3‐year and an interday study
Source: Magn Reson Med. 2025 Mar 13;94(2):588–601. doi: 10.1002/mrm.30495 (PMC12137785; doi:10.1002/mrm.30495)
Supplement: Supplementary file 1 — FIGURE S1. (A) Statistical significances obtained from the coefficient of variation (CV) results obtained from different shim methods, scan groups, and tailored kT‐points pulse (TP) configurations. A significant difference between two scan groups is denoted by a white “S” in a green background, while nonsignificant differences are indicated by a white “NS” in a red background. The prefixes “D,” “TP,” and “UP” denote results from default, TP, and universal kT‐points pulse (UP) shim methods, respectively. Additionally, identifiers c1 to c4 correspond to results from TPs in Config.1 to Config.4, whereas suffixes s1 to s4 denote results from Scan 1 to Scan 4 groups, respectively. (B) Values of ICV,∣x‾∣, and d⊥ for each shim method, normalized to the maximum value reported among all shim groups. Top‐left and bottom‐left figures correspond to data obtained from the Scan 3 and Scan 4 data sets. The top‐center and bottom‐center figures correspond to data obtained from the Scan 1 and Scan 3 data sets. Top‐right and bottom‐right figures correspond to data obtained from the Scan 1, Scan 2, and Scan 4 data sets. Figure S2. Three‐dimensional flip‐angle predictions for the human heart using default, tailored‐pulse (TP; Conf. 1) and universal kT‐points pulse (UP) shim methods. The data correspond to all four scans conducted on the sixth subject. Figure S3. Pulse diagram of complex radiofrequency (RF) voltages (magnitude and phase) and three‐dimensional (3D) gradient blips of the universal kT‐points pulse (UP) and tailored kT‐points pulse (TP) method (all configurations) of the sixth subject. The RF voltage of each parallel‐transmit (pTx) pulse was scaled to achieve a nominal flip angle (FA) of 10° in the three‐dimensional (3D) heart region of interest (ROI), allowing for quantitative comparisons of RF voltages between different pulses. [file MRM-94-588-s001.pdf]

Supporting Information

Reproducibility of tailored and universal non-selective excitation pulses at 7T for human cardiac MRI: A 3-year and an inter-day study

Manuel Fernando Sánchez Alarcón[1,2], Sebastian Dietrich-Conzelmann[1], Jean Pierre Bassenge[1,2], Jeanette Schulz-Menger[2], Sebastian Schmitter[1,4,5] and Christoph Stefan Aigner[1,6]

[1]Physikalisch-Technische Bundesanstalt (PTB), Braunschweig and Berlin, Germany

[2]Charité Université Medicine Berlin, ECRC, a cooperation between the Charité Medical Faculty and the Max-Delbrück Center for Molecular Medicine, Berlin, Germany

[3]German Center for Cardiovascular Research (DZHK), partner site Berlin, Germany

[4]Medical Physics in Radiology, German Cancer Research Center (DKFZ), Heidelberg, Germany

[5]Center for Magnetic Resonance Research, University of Minnesota, Minneapolis, MN, United States

[6]Max Planck Research Group MR Physics, Max Planck Institute for Human Development, Berlin, Germany

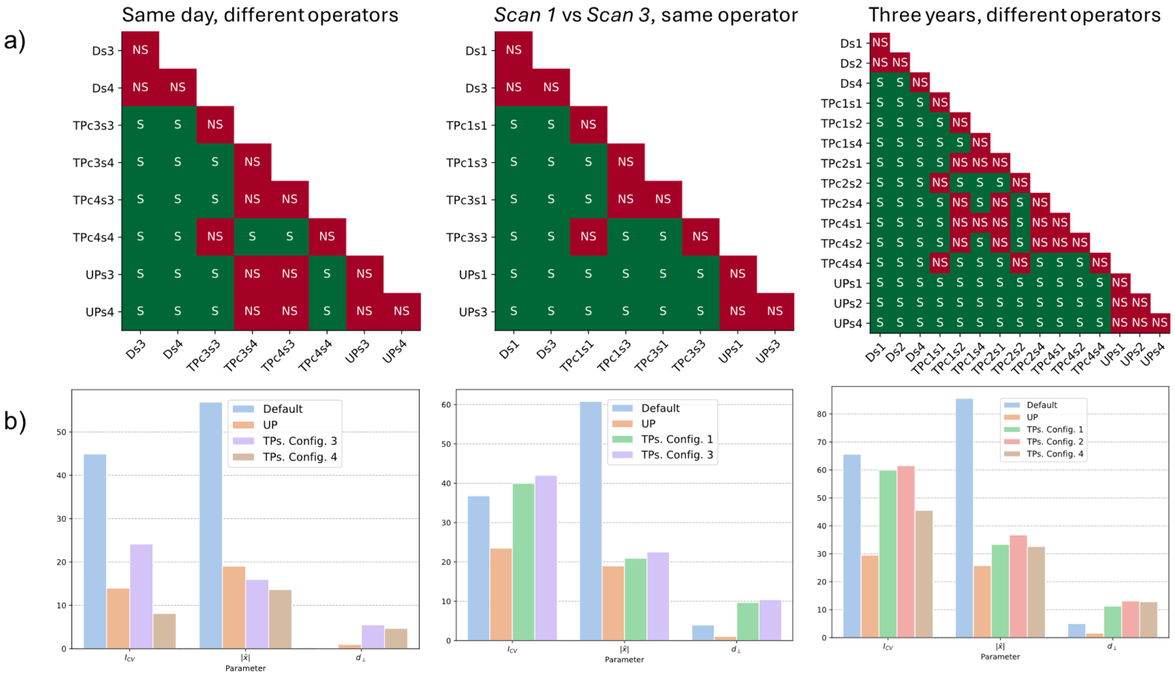

Supporting Information Figure S1: Statistical significances obtained from the CV results obtained from different shim methods, scan groups, and TPs configurations are detailed in (a). A significant difference between two scan groups is denoted by a white “S” in a green background, while non-significative differences are indicated by a white “NS” in a red background. The prefixes D, TP, and UP denote results from Default, TPs, and UP shim methods, respectively. Additionally, identifiers c1 to c4 correspond to results from TPs in *Config.1* to *Config.4*, while suffixes s1 to s4 denote results from *Scan 1* to *Scan 4* groups, respectively. In (b), values of $\boldsymbol{I_{CV}}$, $|\bar{x}|$ and $\boldsymbol{d}_{\perp}$ for each shim method, normalized to the maximum value reported among all shim groups, are presented. Top- and bottom-left figures correspond to data obtained from *Scan* 3 and *Scan* 4 datasets. The top- and bottom-center figures correspond to data obtained from *Scan* 1 and *Scan* 3 datasets. Top- and bottom-right figures correspond to data obtained from *Scan* 1, *Scan* 2, and *Scan* 4 datasets.

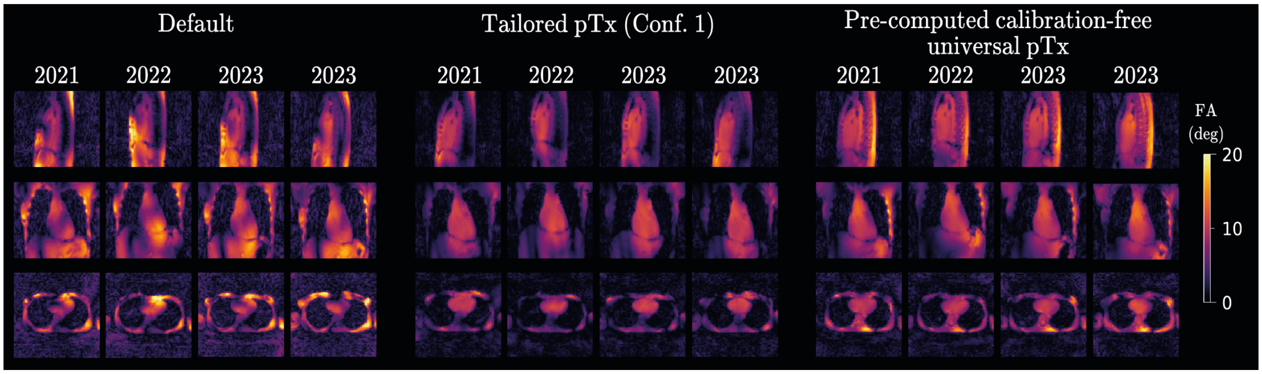

Supporting Information Figure S2: This figure depicts 3D flip angle predictions for the human heart utilizing Default, TPs (*Conf. 1*), and UP shim methods. The data correspond to all four scans conducted on the sixth subject.

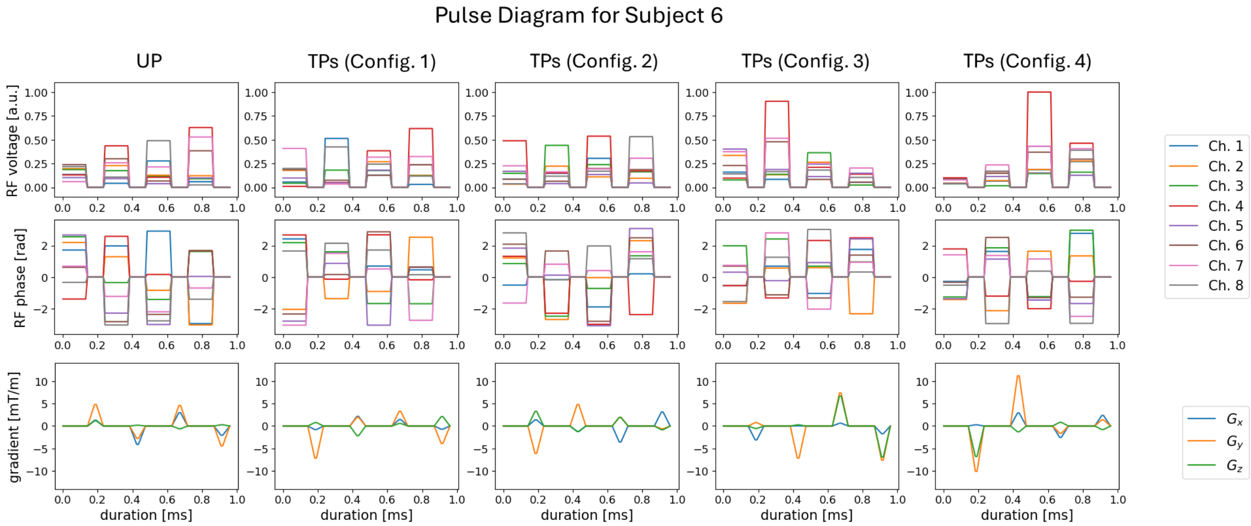

Supporting Information Figure S3: Pulse diagram of complex RF voltages (magnitude and phase) and 3D gradient blips of the UP and TPs method (all configurations) of the sixth subject. The RF voltage of each pTx pulse was scaled to achieve a nominal FA of 10° in the 3D heart ROI, allowing for quantitative comparisons of RF voltages between different pulses.
